# The Peroxisome-Autophagy Redox Connection: A Double-Edged Sword?

**DOI:** 10.3389/fcell.2021.814047

**Published:** 2021-12-16

**Authors:** Hongli Li, Celien Lismont, Iulia Revenco, Mohamed A. F. Hussein, Cláudio F. Costa, Marc Fransen

**Affiliations:** ^1^ Laboratory of Peroxisome Biology and Intracellular Communication, Department of Cellular and Molecular Medicine, KU Leuven, Leuven, Belgium; ^2^ Department of Biochemistry, Faculty of Pharmacy, Assiut University, Asyut, Egypt

**Keywords:** autophagy, disease, hydrogen peroxide, oxidative damage, peroxisomes, pexophagy, thiol-based redox signaling

## Abstract

Peroxisomes harbor numerous enzymes that can produce or degrade hydrogen peroxide (H_2_O_2_). Depending on its local concentration and environment, this oxidant can function as a redox signaling molecule or cause stochastic oxidative damage. Currently, it is well-accepted that dysfunctional peroxisomes are selectively removed by the autophagy-lysosome pathway. This process, known as “pexophagy,” may serve a protective role in curbing peroxisome-derived oxidative stress. Peroxisomes also have the intrinsic ability to mediate and modulate H_2_O_2_-driven processes, including (selective) autophagy. However, the molecular mechanisms underlying these phenomena are multifaceted and have only recently begun to receive the attention they deserve. This review provides a comprehensive overview of what is known about the bidirectional relationship between peroxisomal H_2_O_2_ metabolism and (selective) autophagy. After introducing the general concepts of (selective) autophagy, we critically examine the emerging roles of H_2_O_2_ as one of the key modulators of the lysosome-dependent catabolic program. In addition, we explore possible relationships among peroxisome functioning, cellular H_2_O_2_ levels, and autophagic signaling in health and disease. Finally, we highlight the most important challenges that need to be tackled to understand how alterations in peroxisomal H_2_O_2_ metabolism contribute to autophagy-related disorders.

## Introduction

Autophagy is a conserved catabolic program for the degradation of cytoplasmic components (e.g., dysfunctional organelles, protein aggregates, and non-specific portions of the cytoplasm) within the lysosome (Kaur and Debnath, 2015). Dysregulation of this process has been linked to pathologies such as neurodegeneration, cancer, and diabetes (Kenific and Debnath, 2015; Fang et al., 2019; Muralidharan et al., 2021). Depending on the delivery route of the cytoplasmic material to the lysosome interior, three primary types of autophagy have been recognized in mammalian cells: microautophagy, macroautophagy, and chaperone-mediated autophagy (CMA) (Yim and Mizushima, 2020).

Proteins degraded by CMA contain a KFERQ-like motif that binds to HSC70, a cytosolic chaperone that delivers its cargo to the lysosomal surface for internalization and rapid degradation (Kaushik and Cuervo, 2018) (all protein acronyms are annotated as in the UniProtKB database and the full names can be retrieved in the Glossary). Approximately 30% of all soluble cytosolic proteins contain such a CMA-targeting motif (Chiang et al., 1989), and-after binding of the substrate-chaperone complex to the transmembrane protein LAMP2A (Cuervo and Dice, 2000)—the substrate proteins need to unfold before they cross the lysosomal membrane (Rout et al., 2014). These processes occur in cooperation with a set of cochaperones (Rout et al., 2014; Kaushik and Cuervo, 2018).

In microautophagy, the lysosomal or endosomal membrane is deformed to directly engulf cytosolic materials (Yim and Mizushima, 2020). At the morphological level, cargo can be engulfed through lysosomal protrusion, lysosomal invagination, or endosomal invagination (Oku and Sakai, 2018). At the mechanistic level, many aspects remain to be clarified (Mejlvang et al., 2018; Mesquita et al., 2021). However, this degradation route requires neither LAMP2A nor substrate unfolding (Yim and Mizushima, 2020).

Unlike the former two kinds of autophagy, macroautophagy involves the sequestration of cargo at a distinct site from lysosomes. During this process, a double-membrane structure (the phagophore) is formed (Klionsky et al., 2021). This structure wraps around the cytoplasmic target and creates, upon closure, a separate compartment (the autophagosome). This short-lived organelle subsequently fuses with lysosomes to deliver its content for degradation (Kriegenburg et al., 2018). Given that macroautophagy, hereafter referred to as “autophagy,” is widely recognized as the major removal pathway for organelles in mammalian cells (Klionsky et al., 2021), we focus here on the double-sided redox connection between peroxisomes and this type of autophagy: on one hand, as a major site of intracellular H_2_O_2_ metabolism, peroxisomes have the potential to turn on and tune autophagy, and this process may eliminate dysfunctional peroxisomes and protect the cell from oxidative damage; on the other hand, chronic impairment of peroxisome function may lead to an accumulation of H_2_O_2_ levels that inhibit autophagy, thereby driving a vicious cycle between peroxisome malfunction and cellular redox imbalance.

## The Core Autophagy Machinery

**FIGURE 1:**
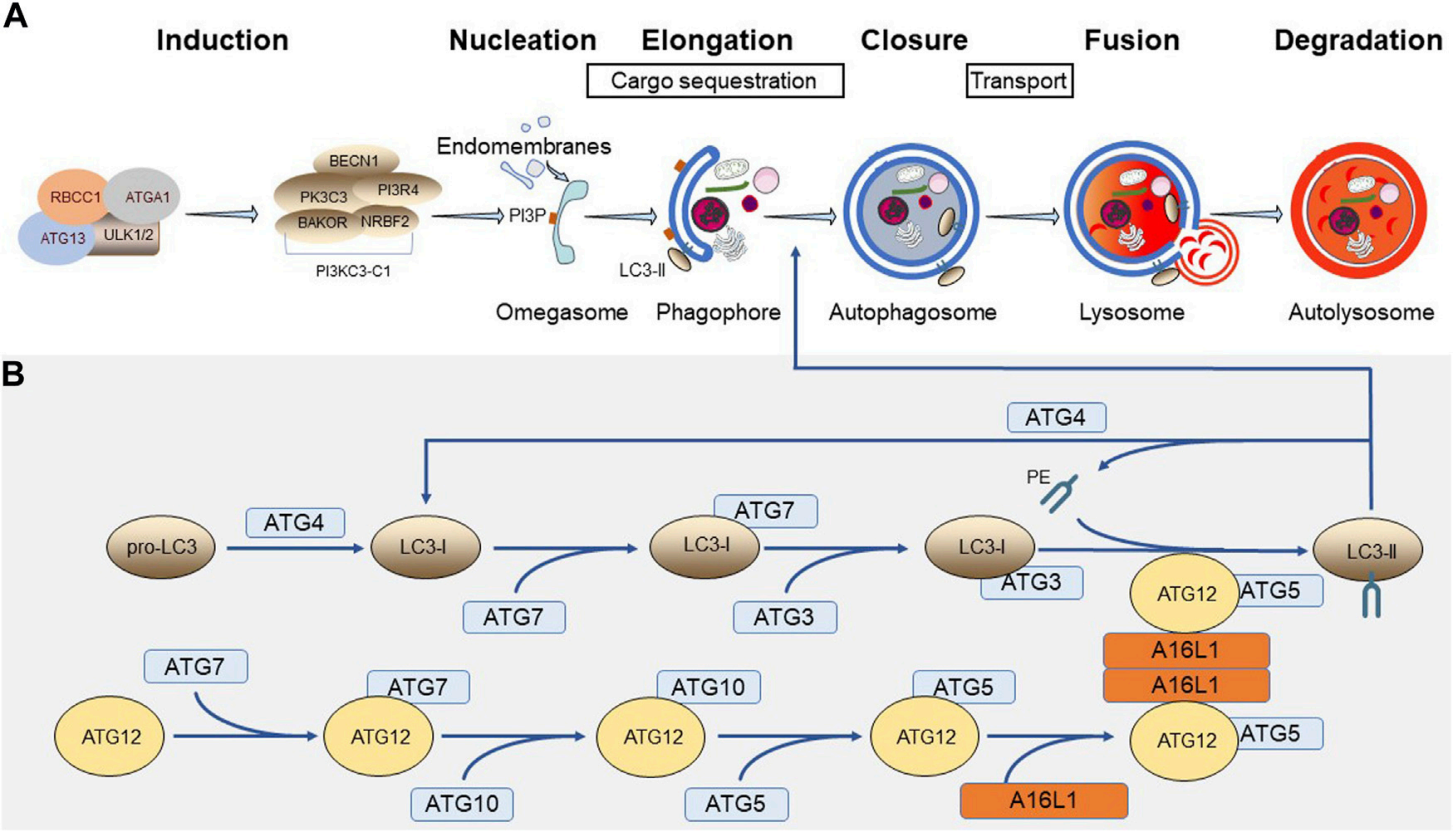
Distinct steps and core components of autophagy. **(A)** Different stages of canonical autophagy. **(B)** Ubiquitin-like conjugation systems involved in phagophore biogenesis.

The canonical autophagy process can be divided into multiple stages, including induction, phagophore nucleation and elongation, cargo sequestration, phagophore closure, autophagosome transport, and cargo degradation via fusion with lysosomes (Figure 1A) (Parzych and Klionsky, 2014; Yim and Mizushima, 2020; Melia et al., 2020). Many of these processes are executed by a dedicated cohort of autophagy-related (ATG) proteins (Feng et al., 2014). Here, we mainly focus on the molecular players involved in phagophore biogenesis (Figure 1B) (Kawabata and Yoshimori, 2020).

The phagophore (or isolation membrane) is a small cup-shaped pre-autophagosomal structure that can originate from endomembranes (e.g., the ER membrane) containing phosphatidylinositol 3-phosphate (PI3P)-enriched subdomains, often referred to as omegasomes (Roberts and Ktistakis, 2013). PI3P is a phospholipid that is mainly generated by the class III phosphatidylinositol 3-kinase complex I (PI3KC3-C1), which is composed of a catalytic subunit (PK3C3), a PI3-kinase regulatory subunit (PI3R4), an allosteric modulator (BECN1), another regulator (NRBF2), and a phagophore targeting subunit (BAKOR) (Hurley and Young, 2017). Activation of the PI3KC3-C1 complex requires the serine/threonine protein kinase complex ULK. This complex, which integrates upstream nutrient and energy signals to coordinate autophagy induction, is made up of either ULK1 or ULK2 and 3 non-catalytic subunits (ATG13, RBCC1, and ATGA1) (Hurley and Young, 2017). Upon induction of autophagy, the ULK1 complex becomes active and translocates to the omegasome, where it exerts its function on PI3KC3-C1 (Mercer et al., 2018).

Elongation of the phagophore requires the concerted action of two ubiquitin-like conjugation systems. Here, it is worth noting that the ATG protein family includes two ubiquitin-like protein members, ATG8/MLP3/GBRL (here denoted as LC3) and ATG12 (Mohan and Wollert, 2018). Following translation, the pro-LC3 proteins first need to be cleaved into their LC3-I counterparts to expose a glycine residue at their C-terminus. This event, which is catalyzed by the cysteine protease ATG4, is necessary to become a substrate for ATG7, an E1-like enzyme that can activate both LC3-I and ATG12. ATG7 in turn transfers its activated ubiquitin-like substrates to ATG3 (in case of LC3-I) or ATG10 (in case of ATG12), two E2-like enzymes. Next, ATG12 is transferred onto ATG5 to form a complex with E3-like activity that, upon interaction with the A16L1 dimer (Lystad et al., 2019), is targeted to the autophagosomal membrane, where it stimulates the ATG3-mediated conjugation of LC3-I to phosphatidylethanolamine (PE) (Martens and Fracchiolla, 2020). This lipidated form of LC3, termed LC3-II, is the active form of LC3 and plays an important role in phagophore membrane expansion, cargo selection, and membrane closure (Li and Zhang, 2019). Note that ATG4 can also delipidate LC3-II to release this molecule from the autophagosomal membrane for reuse (Nakatogawa, 2013).

The closed autophagosome needs to be transported to and fused with the lysosome for digestion. These processes require cytoskeletal filaments, motor proteins, RABs, SNAREs, tethering factors, and lysosomal hydrolases. For a detailed overview of these factors, which are not further elaborated on here, the reader is referred to other dedicated reviews (Nakamura and Yoshimori, 2017; Yim and Mizushima, 2020; Zhao et al., 2021).

## Cargo Receptors for Selective Autophagy

Depending on the nature of the substrate, autophagy can be classified into selective or non-selective (bulk) autophagy (Lamark and Johansen, 2021). In bulk autophagy, portions of the cytoplasm are randomly sequestered, degraded, and recycled to compensate for nutrient deficiencies. Selective autophagy, however, rather serves to eliminate functionally redundant or damaged cytoplasmic components, which are selectively sequestered and degraded as a stress response or quality control mechanism. Examples of selective autophagy processes in mammalian cells include aggrephagy (protein aggregates), ER-phagy (ER), ferritinophagy (ferritin), glycophagy (glycogen), lipophagy (lipid droplets), lysophagy (lysosomes), mitophagy (mitochondria), nucleophagy (nuclear fragments), pexophagy (peroxisomes), ribophagy (ribosomes), xenophagy (bacteria and viruses), and zymophagy (zymogen granules) (Gatica et al., 2018; Gubas and Dikic, 2021).

**FIGURE 2:**
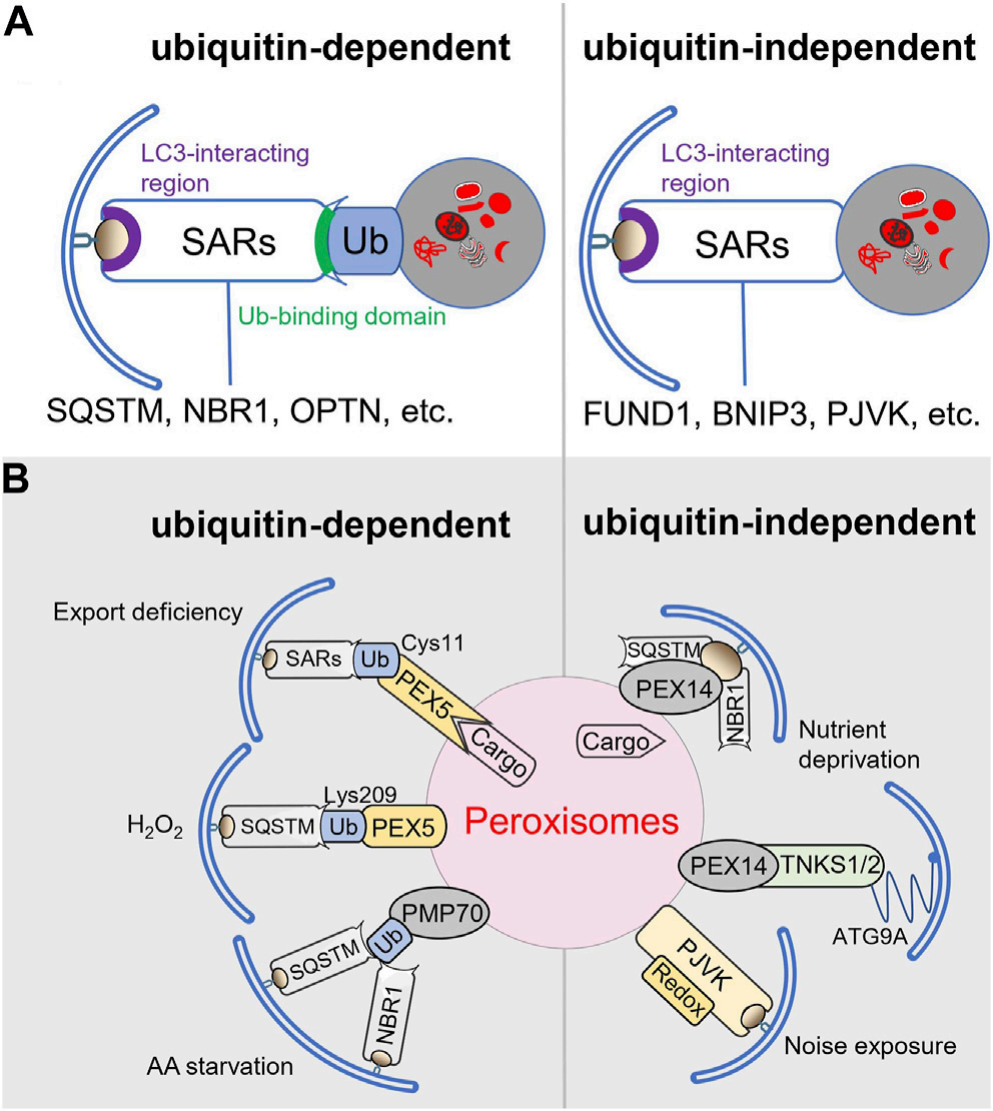
Selective autophagy pathways and pexophagy receptors. **(A)** Types of selective autophagy. **(B)** Cargo receptors involved in selective pexophagy.

Both selective and bulk autophagy utilize the same core autophagy machinery. However, selective autophagy pathways require the additional action of specific autophagy receptors (SARs) including, among others, BNIP3, BNI3L, CACO2, FUND1, NBR1, OPTN, RETR1, and SQSTM (Figure 2A) (Gubas and Dikic, 2021). SARs can act either independently or cooperatively to bridge substrates to phagophores (Kirkin et al., 2009; Cemma et al., 2011). Therefore, they possess a cargo-binding domain as well as an LC3-interacting region. Given that autophagic substrates are frequently ubiquitinated, the cargo-binding domain is often a ubiquitin-binding domain (Kirkin and Rogov, 2019). Specific examples of such canonical SARs include SQSTM, NBR1, OPTN, and CACO2 (Kim et al., 2016). Importantly, the specificity, activity, and stability of most SARs are controlled by a diverse range of post-translational modifications (e.g., phosphorylation, ubiquitination, acetylation) and structural changes (e.g., oligomerization) (Gubas and Dikic, 2021), which may vary within different cell types or under specific environmental conditions.

## Cargo Receptors for Pexophagy

To maintain peroxisome functionality, the superfluous and dysfunctional organelles need to be selectively removed through activation of a specialized form of autophagy, called pexophagy (Nordgren et al., 2013). Currently, there is good evidence that, in mammalian cells, pexophagy can occur through both ubiquitin-dependent and -independent mechanisms (Figure 2B) (Li and Wang, 2021).

Evidence that ubiquitin can indeed function as a self-removal signal for peroxisomes was obtained from an elegant proof-of-concept study showing that heterologous expression of peroxisomal membrane protein (PMPs)-ubiquitin fusion proteins in COS-7 or HeLa cells triggered pexophagy on condition that the ubiquitin moiety was facing the cytosol (Kim et al., 2008). Currently, the autophagy receptors SQSTM and NBR1 are both recognized to participate in this process (Kim et al., 2008; Deosaran et al., 2013; Germain and Kim, 2020). However, given that 1) exogenous expression of NBR1, but not SQSTM, promotes peroxisome clustering and lysosome targeting, 2) SQSTM increases the efficiency of NBR1-mediated pexophagy, and 3) SQSTM is not required for pexophagy upon NBR1 overexpression, their precise roles may differ (Deosaran et al., 2013). In addition, it cannot be ruled out that the relative contribution of NBR1 and SQSTM (or any other SAR) to pexophagy varies depending on the initial stimulus. In this context, it is worthwhile noting that in oxidative stress-induced pexophagy the contribution of SQSTM appears to be more important than that of NBR1 (Zhang et al., 2013; Jo et al., 2020b). Also, for a long time, it was unclear which endogenously ubiquitinated protein was serving as a prime recruitment factor for the SARs involved in pexophagy. Currently, it is common knowledge that PEX5, the cycling import receptor for peroxisomal matrix proteins, plays an active role in this process (Subramani, 2015; Li and Wang, 2021). This association is mainly based on the combined observations that 1) after delivery of its cargo into the peroxisome lumen, the protein is monoubiquitinated on a conserved cysteine residue (Cys11 in human PEX5) in order to be extracted from the peroxisomal membrane by the receptor export machinery (Carvalho et al., 2007; Francisco et al., 2017), 2) peroxisome-associated PEX5 can also be ubiquitinated at Lys209 in response to H_2_O_2_ treatment (Zhang et al., 2015), and 3) conditions resulting in an accumulation of (mono)ubiquitinated PEX5 on the peroxisomal membrane trigger peroxisome removal (Nordgren et al., 2015; Zhang et al., 2015; Lee et al., 2018). Importantly, these findings do not exclude that other ubiquitinated peroxisome-associated proteins may also be involved. Here, it should be noted that, besides peroxisome-associated PEX5, also PMP70 is ubiquitinated during amino acid starvation (Sargent et al., 2016). In addition, it is currently accepted that, under basal conditions, the ubiquitination state of PMPs is maintained at a low level by the peroxisome-associated pool of USP30, a ubiquitin-specific protease (Marcassa et al., 2018; Riccio et al., 2019).

Pexophagy can also take place in a ubiquitin-independent manner. Indeed, LC3-II can be recruited to the peroxisomal membrane through direct or indirect interactions with PEX14 or PJVK (Hara-Kuge and Fujiki, 2008; Jiang et al., 2015a; Li et al., 2017; Defourny et al., 2019). PEX14 is a peroxisomal membrane protein that normally functions as a docking factor for cargo-loaded PEX5 (Fransen et al., 1998), and PJVK is a redox-sensitive peroxisome-associated protein involved in sound-induced peroxisome proliferation in auditory hair cells (Defourny et al., 2019). Under conditions of starvation, where PEX5 is mostly cargo-unloaded, PEX14 can interact with LC3-II, thereby promoting peroxisome degradation (Hara-Kuge and Fujiki, 2008). In a follow-up study, the same authors reported that, upon starvation, PEX14 can also interact with NBR1 and SQSTM1, thereby (most likely) triggering conformational changes in PEX14 and increasing its affinity for LC3-II (Jiang et al., 2015b). Finally, large-scale protein-protein interaction studies have uncovered that PEX14 can also interact with TNKS1 and TNKS2, which were subsequently demonstrated to localize to peroxisomes and promote pexophagy under amino acid starvation conditions (Li et al., 2017). Given that TNKS1 and TNKS2 can also interact with ATG9A, an autophagosomal protein that promotes phagophore membrane growth (Nishimura and Tooze, 2020), it was suggested that the PEX14-TNKS/2-ATG9A complex may function as a non-canonical pexophagy receptor upon nutrient starvation (Li et al., 2017).

## Triggers and Signaling Pathways Controlling (Selective) Autophagy

Autophagy serves a critical role in stress response and quality control networks, and imbalances in this process have been recognized as an important contributor to disease states such as neurodegeneration, cardiac ischemia-reperfusion, liver disease, Crohn’s disease, and infections (Murrow and Debnath, 2013). Major cellular stresses that can be linked to autophagy include nutrient and growth factor deprivation, hypoxia, ER stress, DNA damage, and oxidative stress. In the following subsections, we briefly outline how each of these factors can promote autophagy. For more detailed information, we refer the reader to other reviews.

### Nutrient Deprivation

The basic processes underlying autophagy are controlled by complex signaling pathways. Key regulators include the mechanistic target of rapamycin complex 1 (mTORC1) and AMP-activated protein kinase (AAPK), two kinases whose activities respectively inhibit and stimulate autophagy through a coordinated phosphorylation of ULK1 (Egan et al., 2011; Kim et al., 2011): mTORC1 integrates signals from growth factors, nutrients, oxygen levels, and energy status; and AAPK is a sensor and regulator of cellular energy status (Alers et al., 2012). Both mTORC1 and AAPK are master regulators of cell metabolism, thereby linking autophagy to this process (Murrow and Debnath, 2013; Deleyto-Seldas and Efeyan, 2021). However, their actions are in general antagonistic. For example, nutrient deprivation, a condition well-known to induce autophagy, inhibits and stimulates the activities of mTORC1 and AAPK, respectively (Russell et al., 2014). A starvation-induced activation of AAPK also results in phosphorylation and activation of TSC2, a GTPase activating protein that functions as a key negative regulator of mTORC1 through the TSC complex-RHEB signaling axis (Inoki et al., 2003).

### Hypoxia

Hypoxia, a condition in which oxygen availability is limited, is a significant contributor to cell damage in many acute (e.g., ischemic stroke) and chronic (e.g., pulmonary hypertension) disease processes (Lee et al., 2019). Hypoxia can induce autophagy through activation of multiple oxygen-sensitive signaling pathways (Fang et al., 2015). One such pathway involves hypoxia-inducible factors (HIFs). Under normoxic conditions, transcription factors belonging to the HIF protein family are rapidly degraded by the ubiquitin-proteasome system. However, under hypoxic conditions, these proteins are stabilized and translocated to the nucleus to initiate the transcription of genes involved in cellular adaptation and survival, including a set of genes essential for autophagy (e.g., ATG5, ATG7, ATG9A, BECN1, BNIP3, and BNI3L) (Daskalaki et al., 2018). In addition, HIF1 can act as regulator of autophagy by altering the expression levels of genes involved in glucose metabolism (Kierans and Taylor, 2020). Finally, autophagy can also be induced in a HIF-independent manner through hypoxic stress-induced activation of 1) the MK08 signaling pathway (Frazier et al., 2007), 2) the AAPK/TSC2 pathway (Papandreaou et al., 2008), or 3) the unfolded protein response in the ER (Rouschop et al., 2010; Yang et al., 2019). Note that, under normoxic conditions, ER stress can lead to either autophagy stimulation or inhibition (Rashid et al., 2015).

### DNA Integrity

A third factor that can induce autophagy is diminished DNA integrity caused by, for example, UV-sunlight or metabolically-derived reactive oxygen species (ROS) (Juretschke and Beli, 2021). Such insults trigger a set of DNA damage response signaling pathways that lead to activation of PARP1, FOXO3, ATM, and P53: 1) PARP1 is a predominantly nuclear enzyme that converts NAD^+^ into poly(ADP-ribose), and hyperactivation of this enzyme causes NAD^+^ and ATP depletion, a condition promoting AAPK-mediated autophagy activation (Czarny et al., 2015); 2) FOXO3 is a transcription factor known to control the expression levels of multiple autophagy-related genes, including MLP3B and BNIP3, and binding of FOXO3 to the protein kinase ATM triggers autophosphorylation and activation of the latter protein (Rodriguez-Rocha et al., 2011); 3) activation of ATM triggers the initiation of a phosphorylation cascade that regulates the activity of various downstream targets, including AAPK and P53 (Rodriguez-Rocha et al., 2011); and 4) phosphorylation of P53, a multifunctional transcription factor, results in a transcriptional upregulation of TSC2 and PTEN, a negative regulator of PI3K signaling (Rodriguez-Rocha et al., 2011). In the end, all these events contribute to suppression of mTORC1 activity.

### Oxidative Stress

Nutrient and growth factor deprivation, hypoxia, ER stress, and DNA damage can all be linked to perturbations in the cellular redox balance, another autophagy-modulating factor (Li et al., 2015; Sedlackova and Korolchuk, 2020). Indeed, physiologically relevant oxidants such as H_2_O_2_ can oxidatively modify proteins that are directly or indirectly involved in autophagy regulation and execution, thereby potentially effecting their localization, binding affinities, and/or activities (Sedlackova and Korolchuk, 2020). For example, exposure of cells to H_2_O_2_ can 1) upregulate the transcriptional expression of BECN, BNIP3, BNI3L, MLP3C, and SQSTM through activation of HIF1, P53, FOXO3, NFKB, and NF2L2 (Li et al., 2015), 2) activate PI3K signaling through inactivation of PTEN (Koundouros and Poulogiannis, 2018), 3) suppress mTORC1 activity through activation of ATM and AAPK (Wible and Bratton, 2018), and 4) oxidize and inhibit ATG4 (Wible and Bratton, 2018). For the underlying molecular mechanisms and physiological consequences, we refer the reader to *Redox Regulation of Autophagy*. Importantly, although it is generally thought that oxidative stress always induces autophagy as part of a cellular safeguard mechanism to limit oxidative injury, also other critical factors (e.g., the amount of ROS, nutrient availability, etc.) determine whether autophagy is effectively induced or suppressed under conditions of oxidative stress (Kma and Baruah, 2021).

## Triggers and Signaling Pathways Controlling Pexophagy

**FIGURE 3:**
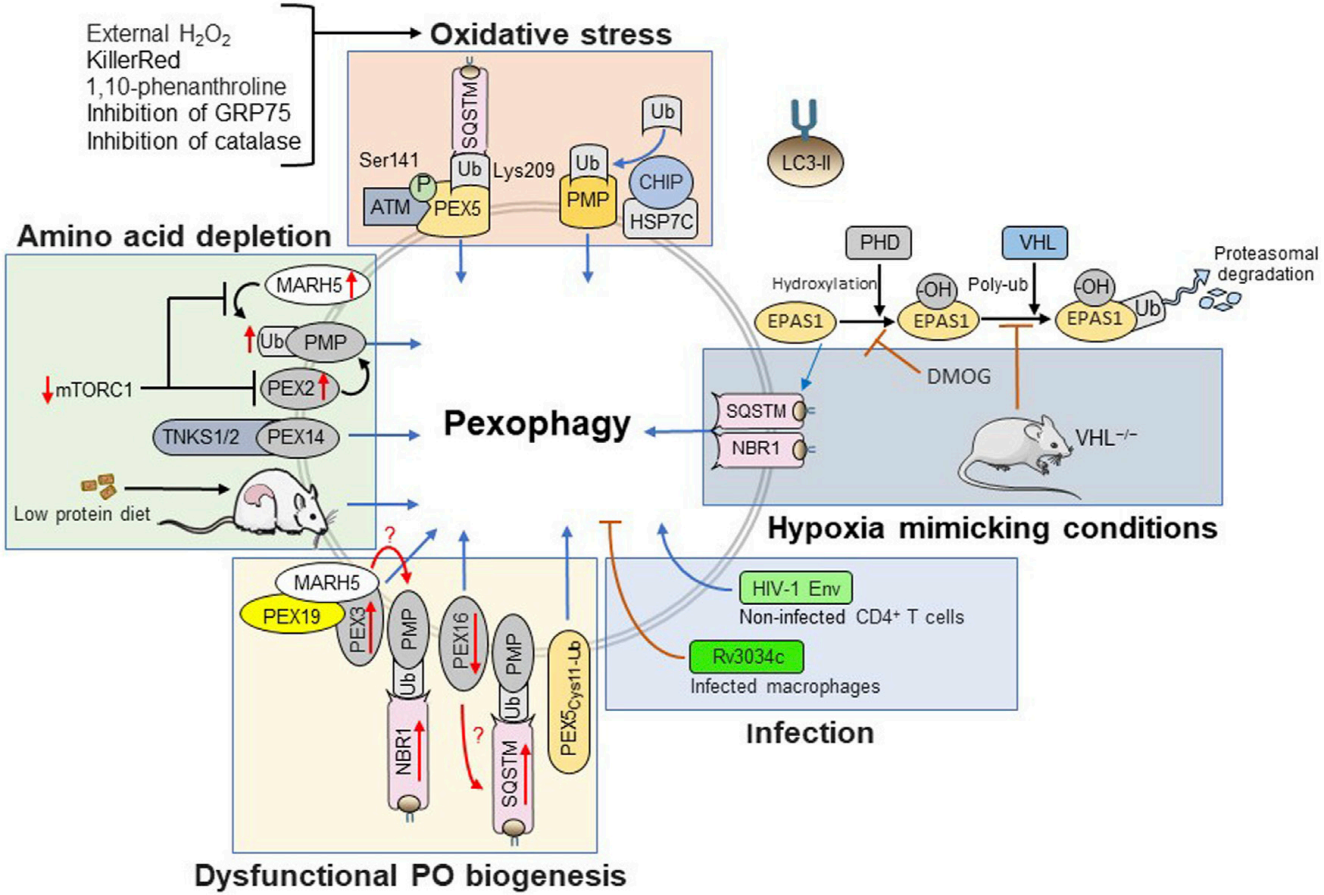
Triggers and signaling pathways controlling pexophagy.

Over the past decade, multiple studies have shown that pexophagy can be triggered by various stress stimuli including amino acid depletion (Li et al., 2017; Dutta et al., 2021), oxidative stress (Zhang et al., 2013; Jo et al., 2015; Zhang et al., 2015; Tripathi et al., 2016; Lee et al., 2018; Defourny et al., 2019; Jo et al., 2020b; Daussy et al., 2020; Dutta et al., 2021), hypoxia (Walter et al., 2014; Mu et al., 2020), viral infection (Daussy et al., 2020), and dysfunctional peroxisome biogenesis (Yamashita et al., 2014; Nordgren et al., 2015; Dahabieh et al., 2021; Wei et al., 2021). In the following subsections, these triggers will be discussed in more detail (Figure 3).

### Amino Acid Starvation

Free amino acids play a vital role in cellular metabolism. Examples include but are not limited to protein synthesis and energy metabolism. Amino acids activate mTORC1 signaling and amino acid starvation suppresses this pathway, thereby inducing autophagy and pexophagy (Germain and Kim, 2020; Deretic and Kroemer, 2021). On one hand, starvation-induced pexophagy can be linked to PEX2, a peroxisomal membrane-associated E3 ubiquitin ligase that is rapidly degraded by the proteasome under basal conditions (Sargent et al., 2016). Upon inhibition of mTORC1 (e.g., during amino acid starvation or upon rapamycin treatment), PEX2 is stabilized and promotes PMP ubiquitination, thereby triggering pexophagy (Sargent et al., 2016). On the other hand, starvation-induced pexophagy can be further enhanced by a PEX14-dependent recruitment of TNKS1/2 onto the peroxisomal membrane (Li et al., 2017). The *in vivo* observation that hepatic peroxisome content is dramatically decreased in rats fed with a low protein diet (Van Zutphen et al., 2014) is in line with the idea that there is indeed a causal link between amino acid starvation and pexophagy. However, direct effectors contributing to pexophagy upon mTORC1 inhibition remain to be identified. Nevertheless, in this regard, it must be noted that a very recent study demonstrated that 1) MARH5, an E3 ubiquitin ligase promoting mitophagy, can also be recruited to the peroxisomal membrane through interaction with the PMP import receptor PEX19 and its membrane docking protein PEX3, 2) recruitment of this E3 ligase to the peroxisomal membrane results in the ubiquitination of PMP70, and 3) MARH5 is playing an important role in mTORC1 inhibition-mediated pexophagy (Zheng et al., 2022). In addition, it is known that prolonged nutrient deprivation causes a significant decrease and increase in the intracellular GSH (Desideri et al., 2012) and H_2_O_2_ levels (Scherz-Shouval et al., 2007b; Chen et al., 2009), respectively.

### Oxidative Stress

Over the last decade, multiple studies have suggested a strong link between oxidative stress and pexophagy, both at the cellular and organismal level. Examples include treatment of Chang liver cells with the chemical 1,10-phenanthroline (Jo et al., 2015), addition of H_2_O_2_ to HepG2 or HEK-293 cells (Zhang et al., 2013), treatment of mouse embryonic fibroblasts with 3-methyladenine (Ivashchenko et al., 2011), loss of GRP75 in neuroblastoma cells (Jo et al., 2020b), peroxisomal KillerRed-mediated ROS production in NIH/3T3 cells (Chen et al., 2020), suppression of catalase expression or activity in serum-starved HepG2 or RPE1 cells (Lee et al., 2018), and prolonged fasting of catalase-deficient mice (Dutta et al., 2021). Note that catalase is a major peroxisomal matrix protein that catalyzes the degradation of H_2_O_2_. Although the underlying molecular details have not yet been fully elucidated, different types of oxidative stressors can induce pexophagy through distinct mechanisms. For example, while it has been claimed that external H_2_O_2_ triggers pexophagy through activation of ATM (Zhang et al., 2015; Tripathi et al., 2016), this kinase appears to be dispensable for the removal of peroxisomes that are oxidatively damaged by activation of peroxisomal KillerRed (Chen et al., 2020). Here, it is important to point out that 1) activation of ATM not only suppresses mTORC1 activity (Wible and Bratton, 2018), but also triggers phosphorylation of PEX5 at Ser 141, an event that subsequently results in its ubiquitination at Lys 209 and the recruitment of SQSTM to the peroxisomal membrane (Zhang et al., 2015; Tripathi et al., 2016), and 2) intraperoxisomal ROS production by KillerRed triggers a HSP7C-mediated recruitment of CHIP, a ubiquitin E3 ligase, onto oxidatively stressed peroxisomes, thereby promoting their selective ubiquitination and autophagic degradation (Chen et al., 2020). Finally, it is worth noting that pexophagy in response to oxidative stress is no mammalian-specific phenomenon. For example, it has been reported that exposure of plant leaves to cadmium induces peroxisomal glycolate oxidase activity (McCarthy et al., 2001), which in turn leads to increased intraperoxisomal H_2_O_2_ levels and pexophagy induction (Calero and Muñoz et al., 2019).

### Hypoxia

Given that peroxisomal respiration can be responsible for up to 20% of the oxygen consumption in tissues such a liver (De Duve and Baudhuin, 1966), it may not come as a surprise that limited oxygen availability (e.g., as a consequence of inadequate vascular networks in solid tumors) and hypoxia mimicking conditions (e.g., upon treatment of cells with HIF prolyl hydroxylase inhibitors such as DMOG) have been found to trigger pexophagy in an EPAS1-dependent manner, at least in certain cell types (Walter et al., 2014; Schönenberger et al., 2015; Mu et al., 2020). EPAS1 is a hypoxia-inducible transcription factor that, under normoxic conditions, is rapidly targeted for proteasomal degradation through hydroxylation by oxygen-sensing prolyl hydroxylases and subsequent recruitment of the von Hippel-Lindau (VHL) ubiquitination complex (Påhlman and Mohlin, 2018). *In vivo* evidence suggested that hypoxia-induced pexophagy involves both NBR1 and SQSTM (Walter et al., 2014). However, although there is evidence that hypoxia can induce ROS formation (Paddenberg et al., 2003; Rathore et al., 2008), the precise mechanisms underlying EPAS1-mediated pexophagy are unclear. Intriguingly, another study reported that peroxisomes are intact and even essential for growth of K562 and HEK-293 cells under hypoxia (Jain et al., 2020). Importantly, these researchers also demonstrated that this phenotype, which was attributed to the organelle’s role in maintenance of membrane fluidity, strongly depended on the medium lipid content and cell seeding conditions (Jain et al., 2020). As such, these seemingly conflicting data may be explained by differences in experimental conditions.

### Viral and Bacterial Infections

Two recent studies have shown that pexophagy can also be modulated by pathogenesis-related proteins. One study demonstrated that the human immunodeficiency virus type 1 (HIV1) envelope glycoprotein (Env) has the potential to induce pexophagy in non-infected bystander CD4^+^ T cells (Daussy et al., 2020). The authors also demonstrated that HIV1 Env can provoke excessive ROS production, a condition that eventually leads to apoptosis thereby very likely contributing to the acquired immunodeficiency syndrome in HIV1-infected patients (Daussy et al., 2020). The other study showed that, upon infection of macrophages, *Mycobacterium tuberculosis* Rv3034c, a putative acetyltransferase, can 1) suppress pexophagy through phosphorylation of mTORC1, an event associated with the down-regulation of pexophagy-associated proteins (e.g., ATG5, NBR1, and SQSTM), and 2) activate peroxisome proliferator activated receptor-γ, a transcription factor that initiates the transcription of peroxisome biogenesis (e.g., PEX3, PEX5, and PEX19) and proliferation (e.g., PEX11B, FIS1, and DNM1L) factors (Ganguli et al., 2020). These changes are likely to favor redox homeostasis, thereby allowing the parasite to avoid ROS-mediated killing (Ganguli et al., 2020). Once again, the precise underlying molecular mechanisms remain unclear.

### Dysfunctional Peroxisome Biogenesis

Another emerging pexophagy trigger is dysregulated PMP or matrix protein import. For example, the peroxisomal membrane proteins PEX3 and PEX16 are essential for PMP assembly, and both overexpression of PEX3 (Yamashita et al., 2014) or silencing of PEX16 (Wei et al., 2021) have been shown to induce pexophagy in an NBR1-and SQSTM-dependent manner, respectively. In addition, conditions leading to an accumulation of (mono)ubiquitinated PEX5 on the peroxisomal membrane (Nordgren et al., 2015; Park et al., 2021b; Dahabieh et al., 2021) can also trigger pexophagy. Note that the latter observation strongly indicates that peroxisome-associated monoubiquitinated PEX5 acts as a key surveillance factor for selective elimination of peroxisomes with a defective PEX5 export machinery (Nordgren et al., 2015; Law et al., 2017; Nazarko, 2017).

## Redox Regulation of Autophagy

Currently, it is widely accepted that autophagy represents a prime mechanism of protection against oxidative damage (Ornatowski et al., 2020; Yun et al., 2020). In addition, autophagic activity is governed by complex redox-mediated signaling pathways that, depending on the context, exert positive or negative regulatory activities at the transcriptional and/or protein level (Scherz-Shouval et al., 2007a; Park et al., 2021a; Redza-Dutordoir and Averill-Bates, 2021; Zhou et al., 2021). In the following subsections, we first briefly explain the mechanisms behind H_2_O_2_ signaling. Next, we elaborate further on how autophagic activity can be directly (e.g., through oxidative modification of autophagy-related proteins) or indirectly (e.g., through oxidative modification of transcription factors or signaling proteins) modulated by H_2_O_2_, the major ROS in redox regulation of biological activities (Lismont et al., 2019b; Sies and Jones, 2020).

### The Concept of H_2_O_2_ Signaling

A main mechanism by which H_2_O_2_ achieves specificity as signaling molecule is through direct oxidation of thiolate groups (RS^−^) in target proteins (Sies and Jones, 2020). These groups can react with H_2_O_2_ to form sulfenic acid (RSOH), an intermediate in inter- or intramolecular disulfide bond formation that–in the presence of high H_2_O_2_ concentrations–can be further oxidized to sulfinic (RSO_2_H) or sulfonic (RSO_3_H) acid. The latter modification is irreversible and causes permanent oxidative damage. Disulfide bond formation can act as a molecular switch to regulate the activity, localization, and stability of redox-sensitive proteins. Importantly, protein thiols with a low reactivity towards H_2_O_2_ can also form disulfide bonds through a redox relay mechanism whereby thiol peroxidases shuttle oxidative equivalents from H_2_O_2_ to other target proteins (Stöcker et al., 2018).

### H_2_O_2_ as a Modulator of ATG Activity

Accumulating evidence points to H_2_O_2_ as a potent modulator of ATG activity (Lizama-Manibusan and McLaughlin, 2013). For example, it has been demonstrated that the human cysteine proteases ATG4A and ATG4B are direct targets for oxidation by H_2_O_2_, thereby rendering them enzymatically inactive through formation of inter- or intramolecular disulfide bridges (Scherz-Shouval et al., 2007b; Zheng et al., 2020). Given the dual function of ATG4 as pro-LC3 cleavage and LC3-II delipidating enzyme (Figure 1), these activities need to be tightly controlled to ensure LC3-I lipidation and autophagy progression when cells are exposed to oxidative insults. To cope with this dual role, it has been proposed that 1) the oxidative modification of ATG4 is mainly taking place at autophagosomes in close vicinity to H_2_O_2_-generating platforms such as mitochondria (Scherz-Shouval et al., 2007b), and 2) the ATG4-dependent cleavage of pro-LC3 into their LC3-I counterparts is more efficient than LC3-II deconjugation (and thus less impacted by partial inhibition of ATG4 activity) (Wible and Bratton, 2018). Nevertheless, although disulfide-bonded ATG4 can be efficiently reduced by the thioredoxin system, it can be expected that harsh or long-term exposure to oxidative stress will eventually fully inhibit ATG4 activity, thereby rather blocking than inducing LC3 lipid conjugation and autophagy (Wible and Bratton, 2018). Besides ATG4, also ATG3, ATG7, and ATG10 have been demonstrated to be redox-sensitive (Filomeni et al., 2010; Frudd et al., 2018). Under basal conditions, ATG3 and ATG7 form inactive thioester-bonded complexes with LC3; upon stimulation of autophagy, ATG3 and ATG7 become active and dissociate from LC3, thereby freeing their catalytic thiols; and under oxidative stress conditions, the non-LC3-shielded thiols in ATG3 and ATG7 form intermolecular disulfide linkages, thereby preventing LC3 lipidation, autophagosome maturation, and autophagy (Frudd et al., 2018). In analogy, it can be expected that the catalytic cysteine of ATG10, another E2-like enzyme, displays a redox-sensitive behavior (Filomeni et al., 2010). However, despite the observation that ATG10 is sensitive to oxidation by H_2_O_2_, this has apparently no impact on the conjugation of ATG5 to ATG12, at least not under the conditions tested (Frudd et al., 2018).

### H_2_O_2_ as a Modulator of SARs Activity

Another important redox-regulated protein in autophagy is the autophagy receptor SQSTM. This protein can undergo self-polymerization through intermolecular disulfide bond formation, thereby facilitating cargo selection and degradation through high-avidity binding to LC3-II on nascent autophagic membranes (Cha-Molstad et al., 2018). The formation of such disulfide-linked conjugates is promoted by oxidative stress conditions, thereby activating prosurvival autophagy (Carroll et al., 2018). Whether or not the other SARs have the capacity to form similar disulfide-linked complexes, remains to be established.

### H_2_O_2_ as a Modulator of Transcriptional Autophagy Regulation

H_2_O_2_-induced posttranslational modifications can also modulate the stability, subcellular localization, and/or activity of many transcription factors (Marinho et al., 2014; Li et al., 2015). Here, we briefly summarize the main impact of H_2_O_2_ on HIF1A, P53, NF2L2, and FOXO, all of which have been implicated in autophagy regulation. For more details regarding the complex molecular mechanisms involved in the adaptive responses, we refer the reader to the references cited. HIF1A is the main driver of transcriptional responses to hypoxia, and H_2_O_2_-induced activation of HIF1A promotes the transcription of BNIP3 and BNI3L, thereby promoting selective mitophagy (Fan et al., 2019; Asgari et al., 2021). The tumor suppressor protein P53 can, depending on its intracellular location (e.g., cytoplasm versus nuclear) and the cellular environment (e.g., normal physiological conditions versus nutrient starvation or hypoxia), modulate autophagy at multiple levels and through diverse mechanisms (Hu et al., 2019). For example, nuclear-localized P53 can directly upregulate the expression levels of TSC2 and the β-scaffolding subunit of AAPK, thereby enhancing autophagy through inhibition of mTORC1 signaling; and cytoplasmic P53 can inhibit autophagosome formation through binding to RBCC1, an A16L1 interactor and component of the ULK complex (Hu et al., 2019). NF2L2 is a transcription factor that controls the expression of genes containing an antioxidant response element in their promoter, such as SQSTM (Puissant et al., 2012). H_2_O_2_ can enhance the expression, stability, and nuclear localization of NF2L2 through sequestration of oxidized KEAP1, a thiol-rich protein that promotes the continuous ubiquitin-mediated degradation of NF2L2 under basal conditions (Li et al., 2015; Yin et al., 2015). Finally, H_2_O_2_ can also activate FOXO transcription factors, which stimulate the transcription of LC3, BNIP3, and ATG12 (Sengupta et al., 2009; Li et al., 2015; Deng et al., 2021a).

### H_2_O_2_ as a Modulator of Autophagy Signaling Pathways

Finally, H_2_O_2_ can also indirectly promote or inhibit autophagy via modulation of the AAPK, PI3K, and mitogen-activated protein kinase (MK) signaling pathways. Importantly, the outcome is context specific. For example, 1) exposure of HEK-293 cells to H_2_O_2_ results in S-glutathionylation of Cys299 and Cys304 (likely Cys297 and Cys302 in UniProt ID P54646) in AAPK2, a catalytic subunit of AAPK, and 2) these oxidative modifications stimulate AAPK activity through release of the autoinhibitory domain from its catalytic core, even under non-ATP depleting conditions (Zmijewski et al., 2010). On the other hand, oxidation of Cys130 and Cys174 has been reported to interfere with AAPK activity under energy starvation conditions, at least in mouse cardiomyocytes (Shao et al., 2014). For PI3K, it was shown that the α- and β-catalytic subunits respectively inhibit and promote autophagy in response to moderate and high levels of ROS (Kma and Baruah, 2021). The α-subunit inhibits autophagy through activation of AKT, a serine/threonine kinase that activates mTORC1 activity and arrests autophagic gene expression; and the β-subunit promotes autophagy through stimulating the activities of PI3KC3-C1 and FOXO (Kma and Baruah, 2021). High levels of ROS can also potentiate the PI3K/AKT pathway through inactivation of PTEN, a phosphatase that counteracts PI3K signaling through dephosphorylation of phosphatidylinositol (3,4,5)-trisphosphate to phosphatidylinositol (4,5)-bisphosphate (Koundouros and Poulogiannis, 2018).

MKs are a group of ROS-regulated serine-threonine protein kinases that play a role in diverse cellular processes (Son et al., 2011), including autophagy (Cagnol and Chambard, 2010; Zhou et al., 2015; He et al., 2018). This class of kinases can be grouped in three subclasses: the extracellular signal-regulated kinases, the c-jun N-terminal kinases, and the p38 kinases (Son et al., 2011; Sui et al., 2014). In general, these MKs can be activated by various oxidative stressors, including H_2_O_2_ (Son et al., 2011), and this subsequently triggers the initiation of complex signaling cascades that eventually modulate, among other processes, autophagic activity. For example, ROS-induced activation of the extracellular signal-regulated kinase pathway can induce adaptive and protective autophagy-associated responses in urinary protein-irritated renal tubular epithelial cells (Deng et al., 2021b); c-jun N-terminal kinase activation can enhance autophagy through 1) upregulation of LC3 (Sun et al., 2011) and DRAM1, a damage-regulated autophagy modulator (Lorin et al., 2010), and 2) the liberation of BECN1 from BCL2/B2CL1 (Zhou et al., 2011); and ROS-induced activation of p38 can induce the expression of various autophagy-related genes (McClung et al., 2010).

## The Peroxisome-Autophagy Signaling Axes

**FIGURE 4:**
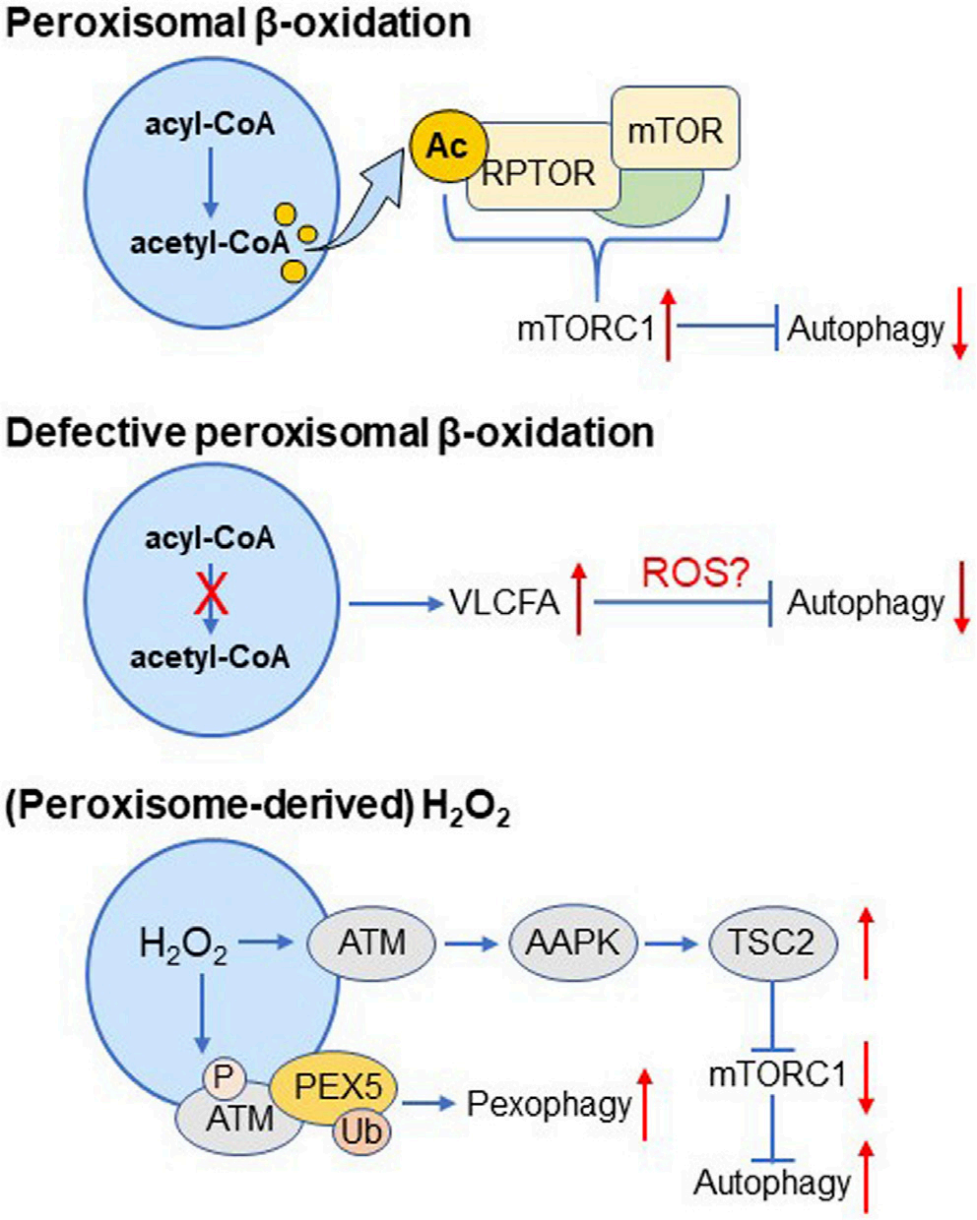
The peroxisome-autophagy signaling axes.

Peroxisomes act as master regulators of cellular lipid and H_2_O_2_ metabolism (Van Veldhoven, 2010; Lismont et al., 2015), and emerging evidence hints changes in peroxisomal lipid or H_2_O_2_ metabolism have the potential to modulate autophagic activity (Figure 4). Specifically, peroxisomal β-oxidation-derived acetyl-CoA can downregulate autophagy by enhancing acetylation of the mTORC1 subunit RPTOR, a process driving mTORC1 activation (He et al., 2020); defects in peroxisomal β-oxidation can suppress autophagy through redox imbalances associated with an accumulation of very-long-chain fatty acids (VLCFAs) (Fourcade et al., 2015; Launay et al., 2015); and (peroxisome-derived) H_2_O_2_ can activate the peroxisomal pool of ATM (Tripathi et al., 2016), an event that enhances 1) autophagic flux through AAPK-TSC2-mediated suppression of mTORC1 activity, and 2) pexophagy through phosphorylation and subsequent ubiquitination of the peroxisome-associated pool of PEX5 (Zhang et al., 2013; Tripathi and Walker, 2016). At first sight, these findings appear somewhat paradoxical. However, this may highlight the complexity of the peroxisome-autophagy signaling axis and point to the importance of other factors. For example, it is well known that disturbances in peroxisomal fitness are intrinsically linked to mitochondrial redox imbalances (Fransen et al., 2017). In addition, we recently found that peroxisomes with a dysfunctional H_2_O_2_ metabolism are not necessarily predisposed to pexophagy, even though peroxisome-derived H_2_O_2_ has the potential to oxidize redox-sensitive cysteine residues in PEX5, PTEN, NFKB1, TF65, and FOXO3 (Lismont et al., 2019a), all proteins whose activities can be linked to pexophagy or autophagy regulation (Subramani, 2015; Füllgrabe et al., 2016; Koundouros and Poulogiannis, 2018). Importantly, given that H_2_O_2_ shows a Janus-faced effect on autophagy (see *Redox Regulation of Autophagy*), it remains to be investigated whether the observed oxidative modifications lead to autophagy stimulation or inhibition. In addition, it still must be clarified if and to which extent other redox-sensitive autophagy-related proteins can act as a target of peroxisome-derived H_2_O_2_.

## The Oxidative Stress-Pexophagy Signaling Axes

Oxidative stress is generally considered as one of the key mediators of cellular aging, a process that coincides with a decline in autophagic activity (Leidal et al., 2018) and a build-up of peroxisomes with a disturbed H_2_O_2_ metabolism (Legakis et al., 2002; Houri et al., 2020). Given that 1) in senescent cells, peroxisomes accumulate excessive amounts of PEX5 on their membranes (Legakis et al., 2002), 2) extraction of PEX5 from the peroxisomal membrane requires monoubiquitination of the protein at Cys11 (Carvalho et al., 2007), 3) Cys11 of human PEX5 functions as a redox switch that modulates the protein’s activity in response to intracellular oxidative stress (Apanasets et al., 2014), and 4) excessive peroxisomal H_2_O_2_ production decreases the intracellular levels of the (peroxisome-associated) PEX5_Cys11-ubiquitin_ thioester conjugate (Lismont et al., 2019a), these findings point to an oxidative stress-induced decrease in PEX5-mediated pexophagy. On the other hand, dysfunctional autophagy/pexophagy will also lead to accumulation of SQSTM, a SAR linking autophagy and NF2L2 signaling through KEAP1 sequestration (Jiang et al., 2015a; Bartolini et al., 2018). This in turn enhances the expression of peroxisome proliferator-activated receptor-γ coactivator-1α, a protein whose expression is antioxidant response element-regulated (Gureev et al., 2019) and causes peroxisomal remodeling and biogenesis (Bagattin et al., 2010). As such, oxidative insults and defects in autophagy can lead to an accumulation of oxidatively burned-out peroxisomes, thereby fueling a vicious circle of oxidative injury (Vasko and Goligorsky, 2013).

## Pexophagy and Disease

Pexophagy acts as a global regulator of peroxisome homeostasis and quality control and, as such, it does not come as a surprise that perturbations in this process have been linked to multiple disease conditions. Examples include the peroxisome biogenesis disorders (Nazarko, 2017), cancer (Walter et al., 2014; Dahabieh et al., 2018), lipopolysaccharide-induced acute kidney injury (Vasko, 2016), malnutrition-associated liver steatosis (van Zutphen et al., 2014), diabetes (Chu et al., 2020), noise-induced hearing loss (Defourny et al., 2019), HIV1 infections (Daussy et al., 2020), and neurodegenerative diseases such as Alzheimer’s and Parkinson’s disease (Jo et al., 2020a). In the following paragraphs, we outline these examples in more detail.

In case of peroxisome biogenesis disorders, it was suggested that 1) in patients with mutations in genes coding for proteins constituting the ubiquitin-PEX5 export machinery, the disease phenotype is rather caused by excessive removal of peroxisomes than by defects in the peroxisomal matrix protein import machinery (Nazarko, 2017), and 2) low doses of autophagy inhibitors improve peroxisomal matrix protein import and peroxisome function without compromising cell viability (Law et al., 2017). Unfortunately, these findings could not be confirmed by others (Klouwer et al., 2021).

Both excessive and defective pexophagy have been linked to cancer (Dahabieh et al., 2018). For example, loss of peroxisomes due to enhanced pexophagy leads to metabolic alterations that have been suggested to promote a malignant phenotype in human clear cell renal cell carcinomas (Walter et al., 2014); and high expression levels of PEX6, PEX26 or MTOR, three negative regulators of pexophagy, have been associated with decreased patient survival in diffuse large B-cell lymphoma, lung cancer and melanoma cohorts. In the latter case, interference with the function of these proteins increased pexophagy and thwarted drug resistance in human melanoma and lymphoma cells (Dahabieh et al., 2021).

Pexophagy was also found to protect auditory hair cells against noise-induced oxidative damage (Defourny et al., 2019) and to attenuate lipopolysaccharide-induced acute kidney injury (Vasko et al., 2013; Vasko, 2016). In these conditions, the removal of dysfunctional peroxisomes may serve a quality control function to prevent ROS accumulation. On the other hand, enhanced peroxisome turnover because of HIV1 Env expression (Daussy et al., 2020) or mutations in GRP75 (Jo et al., 2020b) sensitize, respectively bystander CD4^+^ T lymphocytes and neuronal cells to oxidative injury, thereby potentially contributing to viral spreading and the progression of Parkinson’s disease. Finally, severe malnutrition-induced pexophagy contributes to hepatic mitochondrial dysfunction (Van Zutphen et al., 2014), and short-term inhibition of pexophagy benefits the health of pancreatic β-cells through elevation of ether phospholipid biosynthesis and by counteracting depletion of n-3 polyunsaturated fatty acids after fat-feeding (Chu et al., 2020).

## Conclusion and Perspectives

Pexophagy is a complex cellular process that is tightly regulated at multiple levels and by distinct stimuli. The data presented in this review support the view that changes in the intracellular redox state have the potential to balance this process through activity modulation of autophagy-related proteins, transcription factors, kinases, phosphatases, and PEX5. An increasing number of studies started to examine the relationship between peroxisomal H_2_O_2_ emission and pexophagy, with a focus on the role of peroxisome-associated ubiquitin-PEX5. Major hurdles that have slowed down these studies include the lack of 1) easily accessible and reliable tools to monitor pexophagy in a dynamic manner, 2) compounds that rapidly and selectively trigger peroxisome degradation, and 3) study models that allow the modulation of peroxisomal H_2_O_2_ production in a time- and dose-dependent manner. Here, it is important to highlight that traditional platforms for studying pexophagy mainly focus on immunoblot and (immuno)cytochemistry analyses of key autophagy and peroxisome markers, which only provide a snapshot of a dynamic situation. In addition, unlike what is sometimes thought, amino acid starvation-induced pexophagy is a non-selective process, as also other kinds of cargo (e.g., portions of the cytosol, endoplasmic reticulum, and mitochondria) are sequestered during this type of “metabolic” autophagy (Deretic and Kroemer, 2021; and references therein). Furthermore, although there is mounting evidence that disturbances in peroxisomal H_2_O_2_ metabolism can trigger pexophagy, some frequently cited key experiments have been carried out by treating cells with external H_2_O_2_ (Zhang et al., 2013), a condition incomparable with intraperoxisomal H_2_O_2_ production (Lismont et al., 2021). As such, the recent development of 1) a genetically modified human cell line in which the intraperoxisomal production of H_2_O_2_ can be selectively modulated in a dose- and time-dependent manner (Lismont et al., 2019a), 2) a peroxisome-targeted variant of mKeima, a pH-sensitive red fluorescent protein suitable for imaging pexophagy in cellulo (Marcassa et al., 2018; Jo et al., 2020b), and 3) new fluorescent probes for *in vitro* and *in vivo* quantification of H_2_O_2_ (Ye et al., 2020), offers new opportunities to dynamically monitor (e.g., by flow cytometry) and study pexophagy flux in living cells in response to controlled fluctuations in peroxisomal H_2_O_2_ levels.

Despite the tremendous progress made in recent years, additional work is needed to better understand the peroxisome-autophagy redox connection and to sort out the exact nature of the mechanisms underlying the seemingly contradictory observations regarding the role of amino acid starvation, oxidative stress, and hypoxia in pexophagy regulation. Questions that deserve further research include but are not limited to: Which proteins with an established role in autophagy regulation are direct or indirect targets of peroxisome-derived H_2_O_2_? How do the corresponding oxidative modifications affect the activities of these proteins? Are the cellular responses induced dose-, time-, and cell type-specific? Do the *in vitro* studies recapitulate the *in vivo* situation? Obtaining answers to these questions will not only help us to unravel the molecular mechanisms underlying the Janus-role of pexophagy in health and disease, but also aid researchers to screen for pharmacological pexophagy regulators that can be used in a clinical setting to compensate for genetic and age-related changes in peroxisome homeostasis.

## Glossary

AAPK: AMP-activated protein kinase
ATG: autophagy-related protein
ATM: ataxia telangiectasia mutated
BAKOR: beclin 1-associated autophagy-related key regulator
BCL: B-cell CLL/lymphoma
B2Cl: Bcl-2-like protein
BECN: beclin
BNIP: ATM, ataxia telangiectasia mutated; BCL2/adenovirus E1B 19 kDa protein-interacting protein
BNI3L: BCL2/adenovirus E1B 19 kDa protein-interacting protein 3-like
CACO: calcium-binding and coiled-coil domain-containing protein
CHIP: C-terminus of Hsc70-interacting protein
CMA: chaperone-mediated autophagy
DMOG: dimethyloxalylglycine
DNM1L: dynamin-1-like protein
DRAM: DNA damage-regulated autophagy modulator protein
Env: envelope glycoprotein
EPAS: endothelial PAS domain-containing protein
ER: endoplasmic reticulum;
FIS: fission protein
FOXO: forkhead box protein
FUND: FUN14 domain-containing protein
GRP: stress-70 protein
HIF: hypoxia-inducible factor
HIV1: human immunodeficiency virus type 1
HSP7C: constitutive heat shock protein 70
HSC70: heat shock cognate 70 kDa protein
HSPA: heat shock protein family A
KEAP: Kelch-like ECH-associated protein
LAMP: lysosome-associated membrane glycoprotein
LC3: microtubule associated protein 1 light chain 3
MARH5: membrane-associated RING-CH protein V
MK: mitogen-activated protein kinase
MLP3: microtubule-associated proteins 1A/1B light chain
NBR: next to BRCA1 gene protein
mTORC: mechanistic target of rapamycin complex
NF2L2: nuclear factor erythroid 2-related factor
NFKB: nuclear factor NF-kappa-B
NRBF: nuclear receptor-binding factor
OPTN: optineurin
P53: cellular tumor antigen p53
PARP: poly (ADP-ribose) polymerase
PE: phosphatidylethanolamine
PEX: peroxin
PI3KC: phosphatidyl 3-kinase complex
PI3P: phosphatidylinositol 3-phosphate
PI3R4: phosphoinositide 3-kinase regulatory subunit
PJVK: pejvakin
PK3C: phosphatidylinositol 3-kinase catalytic subunit
PMP: peroxisomal membrane protein
PTEN: Phosphatidylinositol 3,4,5-trisphosphate 3-phosphatase and dual-specificity protein phosphatase
RAB: RAS-associated binding
RBCC: RB1-inducible coiled-coil protein
RETR: reticulophagy regulator
RHEB: RAS homologue enriched in brain
ROS: reactive oxygen species
RPTOR: regulatory-associated protein of mTOR. SAR, specific autophagy receptor
SNARE: soluble N-ethylmaleimide-sensitive fusion protein attachment receptor
SQSTM: sequestosome
TF: transcription factor
TNKS: poly (ADP-ribose) polymerase tankyrase
TSC: tuberin
ULK: UNC-51 like autophagy activating kinase
USP: ubiquitin carboxyl-terminal hydrolase
VHL: Von Hippel-Lindau
VLCFA: very-long-chain fatty acid
